# Flow Cytometric Assessment of CD26-Positive Leukemic Stem Cells: A Rapid and Valuable Tool in the Diagnosis and Follow-Up of Chronic Myeloid Leukemia

**DOI:** 10.7759/cureus.56944

**Published:** 2024-03-26

**Authors:** Sreerag Kana, Sarah John, Debdatta Basu, Rakhee Kar, Rajesh Nachiappa Ganesh, Biswajit Dubashi

**Affiliations:** 1 Pathology, Jawaharlal Institute of Postgraduate Medical Education and Research, Puducherry, IND; 2 Medical Oncology, Jawaharlal Institute of Postgraduate Medical Education and Research, Puducherry, IND

**Keywords:** flow-cytometry, tyrosine kinase inhibitors, cd26, leukemic stem cells, chronic myeloid leukemia

## Abstract

Context

Chronic myeloid leukemia (CML) is a clonal myeloproliferative neoplasm. Recent studies have suggested that CD26-positive leukemic stem cells (LSCs) circulating in peripheral blood are specific for CML.

Objective

This study was undertaken to determine the proportion of CD26-positive LSCs at diagnosis and its change during tyrosine kinase inhibitor therapy.

Design

This prospective study was conducted on 43 cases of CML at diagnosis. For flow cytometry, peripheral blood cells were stained with CD45, CD34, CD38, CD3, and CD26. A sequential gating strategy with CD45/SSC (side scatter), CD34/SSC, and CD34/CD38 was applied to identify CD45+/34+/38- populations, from which CD26-positive stem cells were identified and compared with controls. Data analysis was done with Kaluza software.

Results

All patients diagnosed with CML were detected with CD26-positive LSCs. The median percentage of CD26-positive CML LSCs was 0.02 with a range of 0.001 to 1.77. None of the control samples showed CD26 positivity. The percentage and absolute count of CD26-positive CML LSCs were reduced after six months of tyrosine kinase therapy in patients with complete hematological remission.

Conclusion

Flow cytometric analysis of circulating CD26-positive CML LSCs is a non-invasive, rapid, and useful tool in the diagnosis and follow-up of CML.

## Introduction

Chronic myeloid leukemia (CML) is caused by a genetic translocation t(9;22) (q34;q11), resulting in the formation of the Philadelphia chromosome (Ph) and the BCR-ABL1 fusion protein [1]. First-line tyrosine kinase inhibitor (TKI) such as imatinib is used for the treatment of CML. Based on the response to imatinib and the risk score at diagnosis, other TKIs are also used for the management. Nearly 50% of patients on TKI attain deep molecular responses and eventually stop the medication after obtaining treatment-free remission (TFR), which is the ultimate goal of TKI therapy [2-5]. However, it has been realized that in many patients, TKI, especially imatinib, is unable to eradicate the disease. This is possibly due to the persistence of leukemic stem cells (LSCs). The deep molecular response may not assure the absence of LSCs as imatinib is not able to eliminate the whole LSCs. CML LSCs are characterized by CD34+/CD38- subset of hematopoietic stem cells (HSCs) with the co-expression of CD26 [6,7]. Studies have demonstrated the presence of circulating LSCs in patients on TFR [8,9]. These minimal residual LSCs can reinitiate the disease in patients on TFR.

CD26 has been found to be consistently present in all CML cases and not present in stem cells in other myeloid neoplasms. Recent studies have strongly suggested that circulating CD26 LSCs in peripheral blood (PB) are specific for CML and flow cytometric evaluation of CD26 expression on LSCs can be used as a new, powerful tool for diagnosis of CML as well as for follow-up [9-13].

This study aims to enumerate CD26-positive LSCs in CML at the time of diagnosis and assess any changes in its levels after treatment with TKI.

## Materials and methods

This prospective observational cross-sectional study included all the newly diagnosed cases of CML as per the standard diagnostic criteria, irrespective of the phase of the disease. Forty-three cases were included in the study with a convenient sampling technique. The sample size was estimated with an expected sensitivity/specificity of CD26 positivity in LSCs for diagnosis of CML as 80% at a 5% level of significance and 15% relative precision. A control group of 10 samples diagnosed with acute myeloid leukemia (BCR-ABL negative), reactive neutrophilic leukocytosis, or stem cell donors on granulocyte colony-stimulating factor (G-CSF) was incorporated in the study to validate the CD26 counting. 

The study was conducted in a tertiary care hospital in Southern India, after obtaining Institute Ethics Committee approval (JIP/IEC/2020/136 dated 19/06/2020). Informed consent was obtained from all the study participants before the recruitment to the study. Blood samples of these patients sent to the hematology lab as part of routine hematological studies were utilized for PB smear preparation and flow cytometry. No additional blood was collected from the patients. PB and bone marrow (BM) reports from the hospital information system were accessed to collect clinical and laboratory data such as complete blood count, blast count, and phase of the disease. Age, gender, and Sokal score were also collected.

After the complete blood count and PB examination, the samples were then analyzed by flow cytometry within 24 hours by using multiparametric flow cytometry (Navios, Beckman Coulter, CA, USA). Analysis with four-color staining was done with stain lyse wash protocol for CD26-positive cell identification. The panel of markers used were CD45 KrO (Clone: J33, Beckman Coulter, CA, USA), CD34 PC7 (Clone: 581, Beckman Coulter, CA, USA), CD38 APC750 (Clone: LS198-4-3, Beckman Coulter, CA, USA), and CD26 PE (Clone: M-A261, Becton Dickinson, NJ, USA). CD3 PB (Clone: UCHT1, Beckman Coulter, CA, USA) was also included in the panel. The CD26 positivity in LSCs was documented. The percentage of CML patients who showed positivity for CD26 in LSCs by flow cytometry was then recorded and compared with the controls. The number of events acquired was 100,000 events.

**Figure 1:**
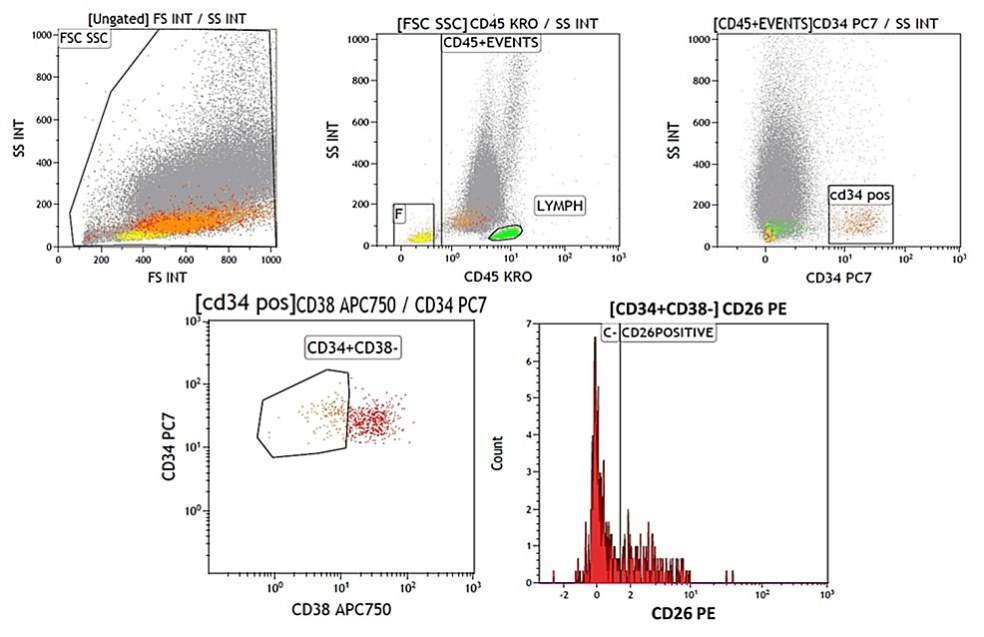
Gating strategy for CD26-positive CML LSC identification CML, chronic myeloid leukemia; LSC, leukemic stem cell.

Evaluation of PB CD26-positive LSCs in CML patients was performed with a sequential gating strategy. To remove debris population, forward scattering (FSC) and side scattering (SSC) light properties were used. Further gating with CD45/SCC was performed on the debris-free population and then a gate was applied on CD34/SSC cells to identify CD45+/CD34+/CD38+ and CD45+/34+/38- compartments. The latter compartment was labeled as LSC. The CD26+ and CD26- stem cell populations were identified in the LSC population. CD3-positive cells/lymphocytes were used as control wherein the divider cutoff based on lymphocyte trough level was used to determine the cutoff on LSCs. The absolute number of CD26+ LSCs in PB samples was calculated as follows: “(WBC count/μL) × (ratio of CD45+/CD34+/ CD38- /CD26+ cells (%))” and expressed as LSC/μL (Figure 1). 

The cases were followed up after imatinib therapy for six months, as part of the routine monitoring protocol. Flow cytometric analysis was again performed on these follow-up cases to see if there was any reduction in circulating CD26+ LSCs after imatinib therapy. Flow cytometry data were analyzed by Kaluza software (Beckman Coulter, CA, USA).

Statistical analysis

Data were entered in Microsoft Excel. The distribution of categorical variables was expressed as frequency and percentage and the continuous data were summarized as mean with standard deviation or median with range or interquartile range based on the normality of distribution. The comparison of the continuous data was done by the Mann-Whitney U test and the Kruskal-Wallis test. The statistical significance of the changes in CD26 positivity over time was assessed by using the Wilcoxon signed-rank test. All statistical analyses were carried out at a 5% level of significance and a p-value <0.05 was considered as significant. Statistical analysis was done with SPSS v20.0 (IBM SPSS Inc., Chicago, IL).

## Results

Basic demographics

Forty-three patients of CML, positive for BCR-ABL1, were labeled as cases, and 10 non-CML cases were taken as controls. These 43 patients had a median age of 39 years ranging from 18 to 70 years, of whom 30 (69.8%) were males and 39 (90.7%) cases had a chronic phase (CP) of CML, while the rest four were in blast crisis. There was no case in the accelerated phase. The basic demographics of the study population are summarized in Table 1. 

**Table 1:** Demographic and hematological details of the 43 patients CML, chronic myeloid leukemia.

Characteristics	Results
Number of participants enrolled, n	43
Median age in years (age range)	39 (18-70)
Phases of CML	
Chronic phase, n	39
Blast crisis, n	4
Sokal score	
High, n (%)	8 (18.6)
Intermediate, n (%)	31 (72.1)
Low, n (%)	4 (9.3)
Hematological parameters	
Mean hemoglobin in g/dL (SD)	8.02 (2.17)
Median WBC count x10^3^/µL (range)	278 (42.6-828.3)
Median platelet count x10^3^/µL (range)	302 (25-1306)

CD26-positive LSCs in CML

The median percentage of CD26-positive cells in CML cases was 0.02 with a range of 0.001 to 1.77. Out of the CD34+/CD38- LSCs, the percentage of CD26-positive cells was 5.04 with a range of 0.05 to 55.94. The absolute count of CD26-positive leukemic cells is calculated as “(WBC count/μL) × percentage of CD26-positive events in CD45+/CD34+/CD38- gate against CD45+ events" and is expressed as CD26-positive LSCs per microliter. The median count of CD26-positive LSCs was 50.72 with a range of 1.95 to 8048.2 cells per microliter (Table 2).

**Table 2:** CD26 expression as percentage and absolute counts in the cases LSC, leukemic stem cell.

CD26 parameters	Median	Range
At diagnosis (n = 43)		
%CD26 LSC	0.02	0.001-1.77
%CD26 LSC in CD34+/CD38- population	5.04	0.05-55.94
Absolute CD26 count	50.72	1.95-8048.2
Follow-up (n = 28)		
%CD26 LSC	0.01	0.001-0.2
Absolute CD26 count	0.7	0.00-15.36

Ten control samples used for the standardization were cases of reactive neutrophilic leukocytosis, acute myeloid leukemia, acute lymphoblastic leukemia, and samples from stem cell donors on G-CSF. None of these 10 cases showed CD26 positivity.

Relationship of CD26-positive LSCs with clinical and laboratory parameters

The median of CD26 count was compared with the Sokal score index and WBC count (WBC ≤ 150 x 10^3^/μL and WBC > 150 x 10^3^/μL). The CD26-positive LSCs count in low, intermediate, and high Sokal index was tested with the Kruskal-Wallis test and there was no statistical difference, χ^2^(2) = 4.05, p = 0.132 with a mean rank CD26-positive LSC count of 23.50 for low risk, 19.81 for intermediate risk, and 29.75 for high risk.

To test the difference in CD26-positive LSCs in WBC count more than 150 x 10^3^ cells/μL compared to WBC count less than or equal to 150x10^3^ cells/μL, the Mann-Whitney test was performed. There was a significant difference in the CD26 counts between the WBC count more than 150x10^3^/μL and WBC count less than or equal to 150x10^3^/μL with a mean rank of 23.87 and 7.80, respectively (Z = -2.690, p = 0.007); however, there was no significant difference in the percentage of CD26-positive LSCs between both groups, Z = -0.748, p = 0.455. A Spearman’s rank-order correlation was run to determine the relationship between the absolute number of CD26-positive LSCs in CML and WBC count. There was a strong, positive correlation between the absolute number of CD26-positive LSCs and WBC count, which was statistically significant (rs value = 0.60, p < 0.001).

CD26-positive LSCs in follow-up cases of CML

Twenty-eight CML-CP cases were available after six months of imatinib therapy and their CD26 status was reassessed. All the patients had achieved complete hematological response by then but their BCR-ABL status at six months of follow-up was not available. The median percentage of CD26-positive LSCs after six months of imatinib therapy was 0.01 with a range of 0.001 to 0.2. The median absolute count of CD26-positive LSCs was 0.7 cells with a range of 0 to 15.36 (Table 2).

A Wilcoxon signed-rank test showed that a six-month treatment with imatinib elicited a statistically significant change in the number and percentage of CD26 LSC in patients with CML (absolute count, Z = -4.2, p ≤ 0.001 and percentage CD26, Z = -2.5, p = 0.011). Thus, the CD26 values and percentage were significantly reduced in those patients who achieved complete hematological remission.

## Discussion

The pathophysiology of CML is due to the formation of the Philadelphia chromosome by the reciprocal translocation of chromosomes 9 and 22 [1]. The resulting oncoprotein, BCR/ABL1, plays a crucial role in the initiation and manifestation of the disease. The management of the disease is done with TKIs, with the assumption that these inhibitors produce a deep molecular response and TFR. The treatment monitoring is done based on the BCR-ABL1 transcript level. It has been realized now that in most patients, imatinib, the first-line TKI, used commonly for management, is not efficient in eradicating CML. This is possibly due to the persistence of LSCs and intrinsic and acquired drug resistance in the residual LSCs [9]. Hence, these LSCs if present in TFR can reinitiate the disease. Hence a marker, specifically identifying the CML LSCs, needs to be identified.

Valent and other researchers reported that a specific type of LSCs in CML called CD34+/CD38-/Lin- co-expresses CD26 and this characteristically distinguishes CML LSCs from normal HSCs more effectively than other previously identified stem cell markers like IL1-RAP, CD25, and CD90 [5,6]. A study in 2014 by Herrmann H et al., on BM samples from CML patients, confirmed the presence of CD34+ CD38-/CD26+ LSCs that are specific to CML. In contrast, CD26+ LSCs were not detected in the BM of healthy individuals or patients with other hematological diseases. The sorted CD26 LSCs from CML patients were confirmed to have the BCR-ABL gene alteration, which is characteristic of CML, in both short-term and long-term colony cells derived from these LSCs. This finding was also confirmed in a mouse model [5].

Culen et al. in 2016 studied 31 patients with CML to see whether CD26+ LSCs could be identified and discriminated from the CD26- HSCs using fluorescence in situ hybridization (FISH) and reverse transcriptase-polymerase chain reaction (RT-PCR) analysis. They reported the variability of the proportion of CD26-positive LSCs and CD26-negative HSCs among the patients and also the fact that CD26 expression robustly discriminated LSCs from HSCs [10]. Bocchia et al. explored the possibilities of quantification of CD26-positive LSCs by flow cytometry in PB samples [8]. They studied 120 PB samples and BM samples for the quantification of CD26-positive LSCs at diagnosis. In the PB, they got a median of 36.9% with a range of 4.2 to 98.8%, while it was 21% with a range of 0.56 to 77.16 in the marrow. They exhibited a strong concordance between CD26% between PB and BM. This meant that PB CD26 levels are suitable for study and one need not perform an invasive test like BM for the same [8]. Raspadori et al. in 2019 studied the feasibility and specificity of the detection of PB CD26-positive LSCs by flow cytometry. Among the 243 patients suspected of CML, 211 were detected with measurable CD26-positive LSCs. All these 211 cases were later found to be BCR-ABL1 positive by FISH and RT-PCR. The median percentage of CD26-positive LSCs in PB was 37.99 with a range of 1.11 to 99.85. None of the 32 samples negative for CD26-positive LSCs showed BCR-ABL1 translocation [12]. Raspadori et al. got fewer CD26-positive LSC absolute numbers in BM than in PS (13.7 vs 15.54), hence advocated that they could be detected directly from PB. Also, LSCs were not affected by the heterogeneity of the CML p210 vs p190 vs p230. Patients on hydroxyurea also showed CD26 positivity, so the drug probably does not affect the CD26 counts [12].

Identifying and assessing CD26-positive LSCs in CML by flow cytometry uses a sequential gating strategy to exclude debris and doublets. The CD45-positive cells were first identified from the CD45/SSC on viable cells. Gate on CD45-positive population by CD34/SSC and CD34/CD38 to identify CD45+/CD34+/CD38+ and CD45+/CD34+/CD38- populations. Then the CD26 expression on LSCs was investigated on CD45+/CD34+/CD38- population [8,10,12-14].

In our study, we found that CD26-expressing LSCs were present in all cases of CML, irrespective of the phase and negative in the control samples used. The median percentage of CD26-positive LSCs was 0.02 ranging from 0.001 to 1.77 and the median absolute CD26 LSC cells was 50.72 cells/μL with a range of 1.95 to 8048.2. CD26 positivity in all phases of CML was in concordance with other studies [11-14]. There appears to be a wide variation in the percentage of CD26-positive cells; some authors have calculated it as the percentage of CD26-positive cells out of the CD34+/CD38- stem cell population [8,11,12], whereas in our study and the study by Sharma et al., it was calculated both from the total WBC count and the CD34+/CD38- stem cells [13].

CD26-positive LSCs have no difference with the different Sokal scores. Culen et al. too didn’t find any relationship between CD26+ cells and Hasford, Sokal, and EUTOS [10]. Similar results were reported by Ebian et al., but they did observe a statistically significant difference after 12 months of TKI therapy between high and intermediate Sokal risk scores [14]. In the present study, CD26-positive LSCs showed a strong correlation with WBC count (rs = 6.0). Similar results were observed by Culen et al. and Ebian et al. [10,14]. Similar to Culen et al., we also classified WBC into two categories (≤150 x 10^3^/μL and >150 x 10^3^/μL) and found that absolute CD26 counts were more in the cases with high WBC counts (Z = -2.690).

Ebian et al. found significantly lower values of CD26 levels after three months and 12 months of treatment (median 0.011 cells/μL) compared to the absolute values at diagnosis (median 17.9 cells/μL) [14]. Bocchia et al. also quantified CD26-positive LSCs in PB of patients with CML on treatment as well as during TFR. They detected CD26+ LSCs in patients on different types of TKI therapy with a median of 0.014 cells/μL. They also reported that CD26-positive LSCs (median number of 0.015 cells/μL) were detected in 66% of patients on TFR with a median duration of 31 months. All of these patients were on first-line TKI at the time of TKI withdrawal. This showed that LSCs persist even during TFR [8]. In our study too, although the CD26 counts were markedly decreased after six months of therapy and complete hematological response than at diagnosis, there was a persistence of detectable CD26-positive LSCs in all patients with a median percentage of 0.01 and a range of 0.001 to 0.2. Pacelli et al., after a median observation time of 33 months in a cohort of 109 patients of CML, confirmed that CD26+ LSCs are detectable at the time of TKI discontinuation and also during TFR. This was also statistically significant for imatinib treatment than nilotinib treatment [9].

Although only 10 non-CML patients were studied as controls, CD26 was not detected in any of them. The majority of the earlier studies have reported CD26 to be consistently negative in non-CML cases [11-13]. This shows the specificity of the CD26 marker to correctly identify the cases with CML.

There have been two published literature from India on CD26 and CML. Both these studies were published in 2022 and were from Northern India [11,13]. Sharma et al. studied 64 patients with CML with a median CD26 percentage of 0.07 with a range of 0.002 to 1.3. All 15 patients who were followed up had detectable amounts of CD26-positive LSCs with a median of 0.003% ranging from zero to 26.79 [13]. Rahman et al. enumerated CD26-positive LSCs from CML patients irrespective of their phase of the disease. All 116 cases of CML had measurable CD26-positive LSCs at diagnosis. Among 105 cases that were in CP with a median of 60.03% with a range of 7.6 to 98.6, four cases were in CML accelerated phase with a median of 38% (range 30 to 49.5) and seven cases were in CML blast crisis with a median of 41.6% (range 9.6 to 97.7) [11]. They also had a good correlation between PB CD26 and BM CD26-positive LSC count although they did in only 12 paired cases. The median range of follow-up of six months showed that those who achieved optimal response had lower CD26 than those who did not. They suggested a cutoff of 70% of CD26-positive LSCs in predicting a suboptimal TKI response [11].

In our study, all patients of CML-CP were treated with TKI and were followed up for six months. After six months of follow-up, the CD26 LSCs were again evaluated in 28 patients. All follow-up patients (n = 28) had attained complete hematological response by then. The follow-up testing with flow cytometry showed a reduced CD26 count with a median absolute count of 0.7 cells/μL with a range of 0 to 15.36. Similar results were reported by Bocchia et al. and Sharma et al. [8,13].

Several studies used BM samples along with PB for CD26-positive LSC enumeration at diagnosis and all of them got a strong concordance [8,11-13] but we didn’t use BM sample for the CD26 enumeration. Follow-up molecular responses were not collected for the data analysis unlike studies [8,13]. These were possibly the two limitations of our study.

## Conclusions

In conclusion, our study adds to the available literature. It highlights the significance of CD26 expression in CML, not only as a potential diagnostic marker but also as a marker during the monitoring of the patients. The heterogeneity of CD26 expression and its correlation with treatment response and TFR underscore the need for further research to fully understand its role in the pathogenesis and management of CML. Incorporating flow cytometry evaluation of CD26 expression into clinical practice may offer a valuable tool for improved diagnosis and monitoring of CML patients.
